# Plasma concentrations of caspofungin in a critically ill patient with morbid obesity

**DOI:** 10.1186/s13054-017-1774-2

**Published:** 2017-08-02

**Authors:** Rafael Ferriols-Lisart, Gerardo Aguilar, Alejandro Pérez-Pitarch, Jaume Puig, Carlos Ezquer-Garín, Manuel Alós

**Affiliations:** 1 Department of Pharmacy, University Clinical Hospital of Valencia, Avda. Blasco Ibañez 17, Valencia, Spain; 2 Surgical Intensive Care Unit, Department of Anesthesiology and Intensive Care, Hospital Clínico Universitario, Valencia, Spain; 3 Health Research Institute, INCLIVA, Avenida Blasco Ibáñez 17, 46010 Valencia, Spain

The aim of the study was to describe the pharmacokinetic behaviour of caspofungin in a critically ill patient with morbid obesity who received doses of caspofungin higher than labelled doses.

A 57-year-old morbidly obese man (BMI = 50 kg/m^2^) was admitted to our ICU after surgical treatment of anastomotic leak in the postoperative period of elective laparoscopic bariatric surgery. The patient was in septic shock, with *Candida* multicolonization and other risk factors of invasive candidiasis. We decided to start antimicrobial treatment, including meropenem, linezolid and caspofungin. With the intention of maximizing the potential effectiveness of antifungal treatment, caspofungin at a dose of 100 mg/day was chosen. The caspofungin dose was calculated using a population pharmacokinetic model [1]. Target AUC/MIC was set at 860 and therefore AUC values above 107 mg*h/L were necessary assuming MIC = 0.125 mg/L [2]. After administration of the first dose, Cpeak was 4.51 mg/L, Ctrough was 0.94 mg/L and AUC was 115.9 mg*h/L. Three days later, Cpeak was 5.97 mg/L, Ctrough was 1.76 mg/L, AUC was 140.4 mg*h/L and AUC/MIC was 1123. The manufacturer recommends caspofungin dose reduction when AUC values are above 210 mg*h/L and, accordingly, drug exposure was considered safe. The patient became apyretic 10 days after caspofungin treatment initiation; this lasted for 14 days without any adverse effects related to this drug.

There are conflicting data on dosing recommendations in obese patients [3]. Payne and Hall [4] found that lower caspofungin AUC was achieved in obese people than in thinner ones, suggesting that dose optimization in heavier patients might improve clinical success rates. If the labelled dose of 70 mg/day had been used in our patient, AUC/MIC would have been 786, below the target AUC/MIC value. Figure 1 shows caspofungin concentrations over time for doses of 100 and 70 mg/day. A dose of 150 mg/day has been recommended in obese patients until simulation studies are completed to provide a bedside dosing formula for caspofungin [4]. There are reports of deterioration of hemodynamic parameters during loading doses in critically ill patients [5]. The monitoring of hemodynamic parameters in these patients is highly recommended. The dose of caspofungin should be adjusted according to both serum caspofungin concentrations and clinical symptoms. However, determination of caspofungin concentrations is performed at only a few laboratories, which makes routine monitoring difficult. This case suggests that caspofungin doses higher than those recommended by the manufacturer may be needed to reach pharmacokinetic/pharmacodynamic targets in ICU morbidly obese patients.

**Fig. 1:**
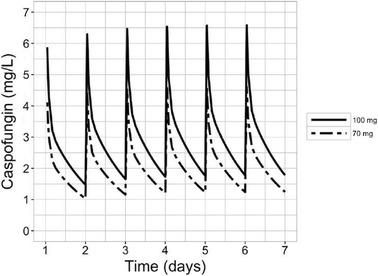
Concentration–time curve for a schedule treatment of 100 mg/day caspofungin versus 70 mg/day in a morbidly obese patient

## Abbreviations

AUC: Area under the concentration-time curve
BMI: Body mass index
Cpeak: Maximum concentration
Ctrough: Trough concentration
ICU: Intensive Care Unit
MIC: Minimum inhibitory concentration required to inhibit the growth of 90% of a microorganism
